# Effect of Anesthetic Modality on Decision-to-Delivery Interval and Maternal–Neonatal Outcomes in Category 2 and 3 Cesarean Deliveries

**DOI:** 10.3390/jcm13247528

**Published:** 2024-12-11

**Authors:** Polona Pečlin, Maja Pavlica, Mirjam Druškovič, Gorazd Kavšek, Ivan Verdenik, Tatjana Stopar Pintarič

**Affiliations:** 1 Department of Perinatology, Division of Obstetrics and Gynaecology, University Medical Centre Ljubljana, 1000 Ljubljana, Slovenia; polona.peclin@kclj.si (P.P.); maja.pavlica@sb-ptuj.si (M.P.); mirjam.druskovic@kclj.si (M.D.); gorazd.kavsek@kclj.si (G.K.); ivan.verdenik@guest.arnes.si (I.V.); 2 Department of Gynecology and Obstetrics, Faculty of Medicine, University of Ljubljana, Šlajmerjeva 3, 1000 Ljubljana, Slovenia; 3 Department of Anaesthesiology and Intensive Therapy, University Medical Centre Ljubljana, 1000 Ljubljana, Slovenia; 4 Institute of Anatomy, Medical Faculty, University of Ljubljana, Vrazov trg 2, 1000 Ljubljana, Slovenia

**Keywords:** decision-to-delivery interval, general anesthesia, spinal anesthesia, epidural anesthesia, neonatal outcomes, maternal outcomes, cesarean delivery anesthesia

## Abstract

**Background/Objectives:** The optimal anesthetic technique for category 2 and 3 cesarean deliveries remains debated, with concerns about impacts on decision-to-delivery interval (DDI) and perinatal outcomes. This study examined the influence of epidural, spinal, and general anesthesia on DDI, surgical and postoperative complications, and neonatal outcomes. **Methods**: This prospective cohort study at a tertiary perinatology center enrolled parturient women undergoing category 2 and 3 cesarean deliveries. Three DDI phases were assessed for each anesthetic modality: transfer time (decision for cesarean section to admission in the operation room), anesthetic time (admission to incision), and delivery time (incision to delivery of the neonate). The surgical procedure time (incision to closure), neonatal (5 min Apgar score, umbilical artery pH/base excess, neonatal intensive care unit (NICU) admission) and maternal (blood loss, surgical and postoperative complications) outcomes were also analyzed for each group. **Results**: There were 215 women (122 category 2 and 93 category 3) included. The use of epidural and general anesthesia was associated with significantly shortened DDI compared to spinal anesthesia (*p* < 0.001). This difference was due prolonged transfer (*p* < 0.05) and anesthetic times (*p* < 0.001), respectively. No cases of umbilical artery pH below 7 were observed in any group. No significant differences were observed in the incidence of umbilical artery pH between 7 and 7.10 or in base excess below −12 nmol/L (*p* = 0.416 and *p* = 0.865, respectively). NICU admission was higher with both general and spinal anesthesia (*p* = 0.021), but mainly due to a higher proportion of preterm births, both before the 32nd week (*p* = 0.033) and between the 32nd and 37th week of pregnancy (*p* < 0.001). General anesthesia was associated with higher maternal blood loss (*p* = 0.026) and a higher rate of postoperative complications (*p* = 0.006). **Conclusions**: In category 2 and 3 cesarean deliveries, general and epidural anesthesia were associated with shorter DDI compared to spinal anesthesia with no differences in neonatal outcomes. General anesthesia was associated with a higher risk of maternal complications compared to neuraxial anesthetic techniques.

## 1. Introduction

The decision-to-delivery interval (DDI) in cesarean sections refers to the time from the obstetrician’s decision to perform the surgery to the delivery of the neonate, or the first neonate in cases of multiple gestations [1]. This interval involves several distinct phases in the complex, multidisciplinary task of preparing for a cesarean section, each with factors affecting the overall timing. To aid in obstetric audits, the DDI can be divided into three critical phases: the time from the cesarean delivery decision to the patient’s admission in the operating room (transfer time), the time from admission in the operating room to the first skin incision (anesthetic time), and the time from the skin incision to the delivery of the neonate (delivery time) [1,2,3]. While all these subintervals can affect procedural efficiency and safety during labor, the transfer time to the operating room and the time needed to achieve adequate anesthesia are key contributors to delays in emergency cesarean deliveries [2,3].

Current guidelines advise the use of standard urgency classifications for operative deliveries involving anesthetists to enhance communication and ensure a rapid, safe, and effective obstetric anesthesia service [4]. The widely adopted four-category classification system by Lucas et al. [5] offers a framework for determining the urgency of a cesarean section, based on the degree of maternal and/or fetal compromise: category 1 involves an immediate threat to the life of the woman or fetus, category 2 includes maternal or fetal compromise that is not immediately life-threatening, category 3 includes the need for early delivery but no maternal or fetal compromise, and category 4 refers to cesarean delivery scheduled at a time to suit the woman and maternity team. While several studies have assessed DDIs for category 1 cesarean deliveries or emergency cesarean sections overall [3,6,7], to the best of our knowledge, none have specifically examined this parameter in the context of category 2 and 3 urgencies. Since the majority of emergency cesarean sections are classified as category 2 or 3, providing targeted information for this group could positively impact many women and enhance the delivery of equitable care.

The urgency of a cesarean section significantly influences the choice of anesthetic technique. For category 1 cesarean sections, rapid sequence induction of general anesthesia is preferred due to its consistently shorter DDI compared to regional anesthetic techniques, assuming no contraindications [8]. While some experienced anesthetists believe that regional anesthesia can match the speed of general anesthesia, the evidence supporting this is both limited and conflicting [3,7,9]. For category 2 and 3 cesarean deliveries, selecting the optimal anesthetic approach is a complex decision marked by a limited understanding of outcome predictive factors [10]. In our obstetric anesthesia practice, general anesthesia is typically used for “crash” (category 1) cesarean sections unless contraindicated. Categories 2 and 3 cesarean deliveries may use spinal, epidural, or general anesthesia, yet there are no definitive guidelines for choosing between these options.

The choice between regional (spinal or epidural) and general anesthesia for cesarean sections has significant clinical implications [11]. Regional techniques offer several benefits including maintaining maternal consciousness and reducing respiratory risks. However, their potential to prolong DDIs in emergencies requires careful consideration. The reported decrease in the use of general anesthesia for cesarean deliveries during the COVID-19 pandemic and the corresponding increase in regional anesthesia call for further investigation into their effects on DDIs and impacts on maternal and neonatal outcomes [6,12]. This study aims to examine the influence of different anesthetic techniques on the DDI, surgical and postoperative complications, and neonatal outcomes in category 2 and 3 cesarean deliveries.

## 2. Materials and Methods

### 2.1. Study Design and Setting

We conducted a prospective, longitudinal cohort study to analyze categorized emergency cesarean deliveries at the Perinatology Department of the Division of Obstetrics and Gynecology, University Medical Centre Ljubljana. This department oversees approximately 5000 deliveries annually, with a cesarean delivery rate of 21%. The study took place from March to October 2021. The focus of the study was to observe and analyze outcomes from routine clinical practice without influencing patient management. Since the study was observational and did not involve any interventions or deviations from standard care, we opted not to register it in a clinical trial registry prior to its initiation. All data were collected retrospectively, anonymized, and handled in compliance with ethical and institutional guidelines to protect participant confidentiality. Since the study did not alter the standard treatment or care provided to the participants, obtaining informed consent for inclusion in the analysis was not required. This approach was also approved by the Republic of Slovenia National Medical Ethics Committee (approval number: 0120-219/2021/3, 23 March 2021). The study was conducted in full compliance with the STROBE guidelines.

### 2.2. Participant Selection

#### 2.2.1. Classification of Cesarean Section Urgency

Women undergoing intrapartum cesarean sections classified as category 2 and 3 urgencies during the study period were included. We excluded category 1 cases, which require obligatory general anesthesia, category 4 cases, where urgent delivery was not indicated, and planned (elective) cesarean procedures, which are performed before the onset of labor. A cesarean section was classified as “elective” if it was planned and scheduled ahead of time, before labor began, instead of allowing labor to start on its own. It was classified as “urgent” if carried out after labor began or due to an unexpected medical complication that made vaginal delivery risky for the mother or baby. Decisions regarding cesarean section were thoroughly documented by a senior obstetrician, adhering to the urgency classification system outlined by Lucas et al. [5]. This classification provides the following predefined urgency categories:

Category 1 (“crash” cesarean section): Indicates an immediate risk to maternal or fetal health, such as severe fetal bradycardia, uterine rupture, cord prolapse, or massive hemorrhage leading to instability.

Category 2: Involves maternal or fetal compromise that is not immediately life-threatening, such as failed instrumental delivery, fetal distress without imminent risk, placental abruption with maternal or fetal compromise, or cephalopelvic disproportion with fetal compromise.

Category 3: Highlights the need for early delivery without immediate compromise, such as cases of cephalopelvic disproportion without fetal compromise, failure to progress in labor, or fetal malposition early in labor.

Category 4: Refers to a delivery planned at a convenient time, which may include situations like failed induction or medical conditions that necessitate a cesarean delivery.

#### 2.2.2. Labour Analgesia Methods

During labor, women in our obstetric unit had the opportunity to choose their preferred method of labor analgesia after discussions with anesthesiologists, obstetricians, and midwives. The available options included remifentanil patient-controlled analgesia (PCA), epidural analgesia, nitrous oxide, and pethidine, ensuring that all participants could access the same analgesic choices.

### 2.3. Anesthetic Techniques

Intrapartum anesthesia for cesarean sections was administered using general, spinal, or epidural techniques. The choice of anesthetic technique was influenced by multiple factors, including the level of urgency, potential contraindications, obstetric considerations, and the preferences of both patients and obstetricians.

#### 2.3.1. General Anesthesia

General anesthesia was administered using a rapid sequence induction approach. Prior to induction, patients received a pre-oxygenation protocol involving 4 to 5 vital-capacity breaths of pure oxygen. Following this, they were given an intravenous dose of either 5 mg/kg thiopental or 2 mg/kg propofol, along with 1 mg/kg of either succinylcholine chloride or rocuronium (1 mg/kg). Endotracheal intubation was carried out once the induction agents took effect, and anesthesia was sustained with sevoflurane in a nitrous oxide/oxygen mixture at a ratio of 60/40. For those who initially received succinylcholine, an additional 0.5 mg/kg of rocuronium was given to ensure continued neuromuscular blockade. Fentanyl, dosed at 3–5 mcg/kg, was administered intravenously following cord clamping and adjusted as needed based on the presence of preexisting epidural analgesia.

#### 2.3.2. Spinal Anesthesia

Spinal anesthesia was performed using a 25-gauge Sprotte needle, inserted at the L3–L4 intervertebral space. A dose of between 7.5 and 11 mg of bupivacaine, combined with 25 μg of fentanyl, was administered. After the injection, patients were positioned supine with a 15° tilt to the left and a 15° Trendelenburg position to facilitate optimal cephalic distribution of the anesthetic agents. Adequate anesthesia was recognized when the upper sensory block reached the T4 level.

#### 2.3.3. Epidural Anesthesia

For patients already receiving epidural analgesia, emergency intrapartum cesarean section anesthesia was achieved by administering an epidural top-up of 2% lidocaine with dosages of up to 20 mL, beginning in the delivery room. Anesthesia was considered adequate if it resulted in an upper sensory block to the T4 level.

### 2.4. Data Collection and Analysis

Data were collected for each urgency group (categories 2 and 3 cesarean deliveries) on maternal clinical and obstetric characteristics, perioperative time intervals, neonatal outcomes, and maternal peri- and postoperative complications. Specific data points for all anesthetic modalities are outlined in Table 1.

Three DDI phases were considered:

1. Transfer time: From the senior obstetrician’s decision to perform a cesarean section to the patient’s admission in the operating room.
2. Anesthetic time: From the patient’s admission to the operating room to the first skin incision.
3. Delivery time: From the skin incision to the delivery of the first fetus.

Additionally, the surgical procedure time, from skin incision to skin closure, was recorded [1,3]. Data analysis was conducted using IBM SPSS Statistics for Windows Version 27.0. Descriptive statistics, ANOVA, and chi-square tests were employed where appropriate. Continuous data with a normal distribution are reported as mean ± standard deviation (SD). One-way ANOVA was used to compare continuous variables, while the chi-square test was used for categorical variables. A *p*-value of <0.05 was considered statistically significant.

The sample size was determined based on the availability of participants within the study period. The sample size was likely to be sufficient to detect meaningful effects, based on the data available from prior studies. We succeeded in enrolling 99% of all urgent cesareans during the study period, so bias was negligible.

We selected 32 weeks of gestation as the cutoff for stratifying preterm cases based on established clinical and developmental milestones. Infants born before 32 weeks are considered very preterm, a group associated with significantly higher risks of severe complications such as respiratory distress syndrome, intraventricular hemorrhage, and necrotizing enterocolitis due to immature organ systems, particularly the lungs and central nervous system. Beyond 32 weeks, neonatal outcomes improve markedly, with lower rates of morbidity and mortality [13,14].

## 3. Results

**Figure 1:**
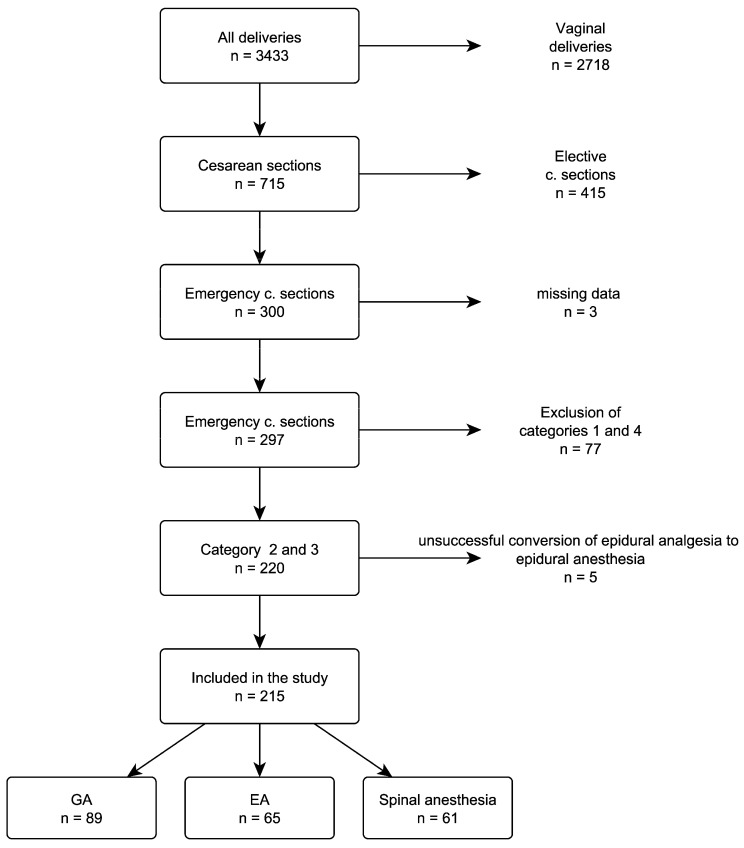
Flowchart of the patient enrollment process. GA = general anesthesia, EA = epidural anesthesia.

During this period, there were 3433 deliveries, with 715 (20.8%) performed via cesarean section. After applying the exclusion criteria, a total of 215 parturient women with cesarean delivery urgency levels of 2 and 3 were included for analysis (Figure 1). In this cohort, 122 (56.7%) of the cesarean deliveries were categorized as category 2. The primary indication for category 2 cesarean deliveries was fetal distress, indicated by a non-reassuring cardiotocography (CTG) reading (44.3%). This was followed by fetal malposition in advanced labor (15.6%), cephalopelvic disproportion with fetal compromise (13.9%), and slow or halted labor progress with fetal compromise (9.8%). The remaining 93 (43.3%) of the cesarean deliveries were classified as category 3. The most common indications in this category were poor labor progress (38.7%), cephalopelvic disproportion without fetal compromise (27.9%), and planned cesarean deliveries that were subsequently considered for urgent delivery due to early onset of labor (12.9%).

**Figure 2:**
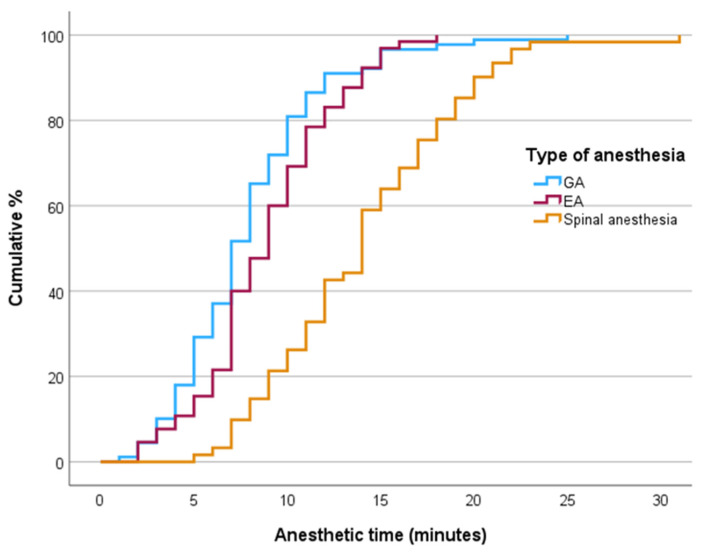
Cumulative Kaplan–Meier curves for anesthesia time (admission to the operation room to first skin incision). The graph depicts the cumulative percentage of category 2 and 3 cesarean deliveries in relation to the time elapsed from entering the operating room to the first incision, stratified by the type of anesthesia used. GA = general anesthesia, EA = epidural anesthesia.

**Figure 3:**
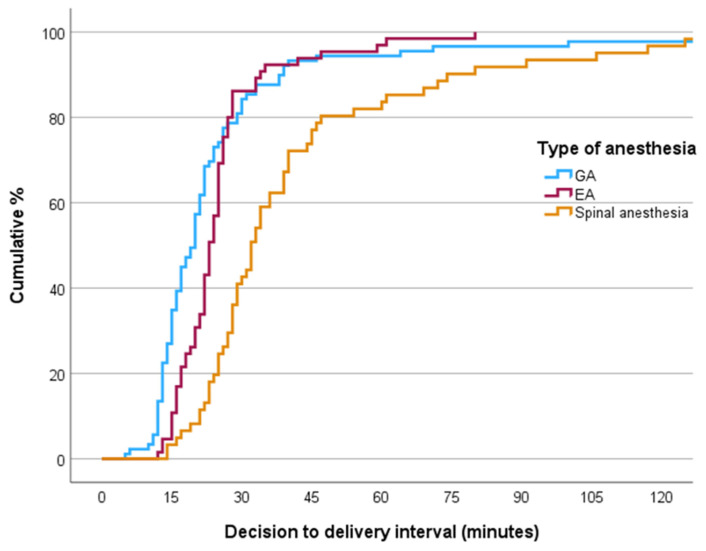
Cumulative Kaplan–Meier curves for DDI (decision-to-delivery interval). The graph depicts the cumulative percentage of categories 2 and 3 cesarean deliveries in relation to the time elapsed from decision to perform cesarean delivery to delivery of the neonate, stratified by the type of anesthesia used. GA = general anesthesia, EA = epidural anesthesia.

The maternal clinical and obstetric characteristics stratified by the type of anesthesia administered during cesarean delivery are summarized in Table 2. There were no significant differences between anesthetic modalities in terms of maternal age, body mass index (BMI), or conception rates by in vitro fertilization (IVF). Epidural anesthesia was predominantly used in nulliparous women (96.9% compared to 62.9% for general anesthesia and 54.1% for spinal anesthesia, *p* < 0.001). A significantly higher proportion of patients in the spinal group had multiple pregnancies compared to the general anesthesia group (11.5% vs. 4.5%, *p* = 0.014). In the epidural group, there were no preterm births. Preterm births before the 32nd week were significantly more common in the general anesthesia group compared to the spinal anesthesia group (10.1% vs. 6.6%, *p* = 0.033), whereas preterm births between the 32nd and 37th weeks were significantly more frequent in the spinal group compared to the general anesthesia group (24.6% vs. 16.9%, *p* < 0.001). Epidural analgesia was successfully converted to epidural anesthesia in 65 (71.4%) parturient women and to general anesthesia in 25 (28.1%) parturient women; 1 (1.6%) parturient received spinal anesthesia (*p* < 0.001). Table 3 details the time intervals for various anesthetic techniques used during cesarean sections and their corresponding maternal and neonatal outcomes, highlighting significant differences among them. The DDIs were significantly shorter for both the epidural anesthesia and general anesthesia groups compared to the spinal anesthesia group (*p* < 0.001). Notably, most cases in the epidural and general anesthesia groups had a DDI of under 30 min (86.2% and 84.3%, respectively), in contrast to only 42.6% in the spinal anesthesia group. The longer DDIs in the spinal group can primarily be attributed to extended decision-to-transfer times (mean 22.5 min for spinal vs. 14.4 min for general and 11.8 min for epidural; *p* = 0.024) and longer transfer-to-incision times (anesthesia time) (mean 14.0 min for spinal vs. 7.9 min for general anesthesia and 8.9 min for epidural; *p* < 0.001). (Figure 2 and Figure 3). However, there were no significant differences among the different anesthesia modalities regarding the duration from the first incision to delivery or in the overall duration of the operative procedure.

No cases of umbilical artery pH below 7 were observed in any group. No significant differences were observed in the incidence of umbilical artery pH between 7 and 7.10 or in base excess below −12 nmol/L (*p* = 0.416 and *p* = 0.865, respectively). Neonatal intensive care unit (NICU) admission rates were significantly higher in the general (19.1%) and spinal anesthesia (19.7%) groups compared to the epidural anesthesia group (4.6%, *p* = 0.021). In both the general and spinal anesthesia groups, there was a significantly higher proportion of preterm births before both the 32nd and 37th weeks, with prematurity being the primary reason for NICU admissions.

Maternal blood loss was significantly higher in the general anesthesia group (mean 525 mL) compared to the epidural group (mean 412 mL, *p* = 0.026). While surgical complications did not differ significantly among the groups, postoperative complications were significantly more frequent following general anesthesia (31.5%) compared to epidural anesthesia (10.8%, *p* = 0.006).

## 4. Discussion

Our study demonstrates that for category 2 and 3 cesarean deliveries, DDI was significantly shorter in both the epidural and general anesthesia groups compared to the spinal anesthesia group, with no differences in neonatal outcomes among the groups. However, the use of general anesthesia was associated with a higher risk of blood loss and postoperative complications. This suggests that while general and epidural anesthesia may facilitate quicker delivery times, the choice of general anesthesia may carry increased risks for maternal health.

Although spinal anesthesia offers advantages such as rapid onset and a dense block, it was associated with longer transfer and anesthetic time. Importantly, there were no significant differences in the surgical subintervals, including incision-to-birth and incision-to-closure durations, indicating that the choice of anesthesia primarily impacts the initial phases of the intrapartum cesarean delivery, while other intraoperative factors may influence subsequent time intervals [15,16]. This suggests that once the surgical procedure commenced, all groups experienced similar operative timeframes. The prolonged decision-to-incision interval observed in the spinal anesthesia group may stem from additional steps involved in its administration, such as patient preparation, positioning, and adherence to aseptic protocols, along with other logistical factors in the labor and operating rooms. Additionally, when spinal anesthesia is chosen, the team may perceive more available time, potentially leading to less urgency in initiating the transfer to the operating room. This highlights the complexity of balancing clinical decision-making with urgency classification. Our findings align with those of McCahon and Catling [3], who reported that the mean time for surgical readiness (anesthetic time) was significantly shorter with general anesthesia (15.4 min) compared to spinal anesthesia (27.6 min) in emergency cesarean deliveries, although their study did not distinguish between different grades of urgency. Similar observations were also made by Wiskott et al. [17].

The target DDIs for categories 2 and 3 cesarean sections are generally less standardized than those for category 1. The National Institute for Health and Clinical Excellence (NICE) recommends prioritizing rapid intervention for unplanned category 1 and 2 cesarean deliveries, establishing target DDIs of 30 min for category 1 and either 30 or 75 min for category 2 as benchmarks for auditing obstetric unit performance [18]. In contrast, the American College of Obstetricians and Gynecologists (ACOG) stipulates a decision-to-incision interval of 30 min for non-elective cesarean deliveries [19]. A common practice that significantly reduces anesthetic time in the operating room is the “topping up” of the epidural catheter, initially placed in the labor room, with a more concentrated local anesthetic of dosages up to 20 mL [20]. Furthermore, general anesthesia typically has a quicker onset compared to regional techniques, enhancing its preference for category 1 deliveries [21,22]. Our analysis revealed that both epidural and general anesthesia were associated with a DDI of under 30 min in over 80% of cases. In contrast, for the spinal anesthesia group, more than 50% of cases exceeded 30 min, with several extending beyond 60 min. These findings are consistent with previous research [3].

Decision-to-delivery intervals exceeding 75 min have been linked to adverse maternal and neonatal outcomes, emphasizing the necessity of minimizing such delays [23,24,25]. However, a large audit report from the UK and a meta-analysis of smaller studies found no significant differences in neonatal outcomes between cases with DDIs of 30 min or less and those exceeding 30 min [23,26]. This lack of correlation between longer DDIs and poorer neonatal outcomes may stem from the “signal-to-noise” issue present in uncategorized emergency cesarean sections, where cases of true fetal compromise are obscured by those with potentially reversible issues [27]. Some experts have suggested favoring general anesthesia for all emergency cesarean deliveries to expedite the DDI and potentially improve neonatal outcomes, given its quicker administration compared to regional anesthetic techniques [21,22]. However, evidence indicates that general anesthesia is an independent risk factor for poor neonatal outcomes in cesarean sections [23,28]. In contrast to our findings, other studies have reported that spinal anesthesia results in shorter times until surgical incision and overall anesthetic duration compared to epidural anesthesia, albeit with a higher incidence of hypotensive episodes [9]. Given the retrospective nature of these studies, caution is warranted in interpreting their findings.

In our study, both general and regional anesthesia groups demonstrated favorable neonatal outcomes. Differences in neonatal outcomes were primarily observed as higher NICU admission rates and lower birth weights in the spinal and general anesthesia groups. These differences can be attributed to the significantly higher proportion of preterm births (both before 32 weeks and between 32 and 37 weeks) in these populations. Notably, epidural anesthesia was not administered in cases of preterm labor, a factor that should be considered when interpreting the results. Importantly, no significant differences in umbilical artery pH were observed between the groups. Additionally, because epidural analgesia is not typically preferred in high-risk obstetric scenarios—such as previous cesarean deliveries, twin gestation, or breech presentation—it is more likely that non-epidural anesthetic techniques are chosen during these complicated deliveries [29].

We found no prior studies specifically examining perinatal outcomes in category 2 and 3 cesarean deliveries concerning intrapartum anesthetic modalities. However, Palmer et al. [28] reported that while general anesthesia provides the fastest operating room to incision interval for category 1 cesarean sections, it is also associated with poorer short-term neonatal outcomes. Specifically, general anesthesia (not the operating room to incision interval) was linked to lower 5 min Apgar scores [28]. The placental transfer of general anesthesia medications can depress the neonatal central nervous system, potentially leading to respiratory complications and reduced Apgar scores [30]. However, the neonatal depression associated with general anesthesia may also reflect the severity of intrapartum fetal compromise necessitating surgery. Our study’s lack of significant differences in Apgar scores across the groups further suggests that the anesthetic technique itself may not be the sole determinant of neonatal well-being. Other factors, such as gestational age and underlying maternal or fetal conditions, also play an essential role.

Furthermore, the use of general anesthesia was associated with higher maternal perioperative blood loss. Similar findings were reported by Hong et al. [31], who demonstrated that, compared to epidural anesthesia, general anesthesia resulted in lower immediate postoperative hematocrit levels and a greater need for blood transfusions. The increased risk of postoperative complications in the general anesthesia group may be attributed to several factors, including the greater physiological stress of surgery, prolonged recovery times due to higher pain intensity, and the necessity for opioids, as well as the potential for airway complications [32,33]. Pregnancy-related physiological and anatomical changes impact oxygenation and airway management, and existing conditions such as obesity or preeclampsia may further complicate general anesthetic care [34]. Additionally, our previous analysis of the same cohort [35] showed that over 60% of parturient women who received general anesthesia had been administered remifentanil patient-controlled analgesia prior to surgery, which is associated with a higher risk of maternal apnea and respiratory depression [36]. Moreover, the increasing popularity of neuraxial techniques in obstetrics has led to a decline in the management of obstetric general anesthesia among trainees, likely contributing to a rise in maternal anesthetic complications such as failed or difficult intubation, hypoxia, and aspiration, which can further elevate peri- and postoperative morbidity [37,38]. In addition to the potential impact of general anesthesia on maternal and perinatal outcomes, various surgical factors—such as obesity, previous cesarean sections, adhesions, and placenta previa—can also increase the risk of significant maternal hemorrhage, iatrogenic injuries, and other complications [16,39].

Meanwhile, although time recommendations for DDIs in practice guidelines continue to serve as a crucial performance metric for obstetric anesthesia services, some argue that without a clear evidence base, these suggested time targets for emergency cesarean sections may act more as potential medico-legal liabilities than as tools to optimize patient outcomes [2,3]. Additionally, these practice guidelines often lack specific definitions and indicators for key time points that define the perioperative intervals, leading to inconsistencies in clinical practice with potential medico-legal ramifications [1]. Indeed, despite its widespread use as an obstetric performance metric, the DDI lacks consistent definitions in the existing literature, particularly regarding its start and endpoints, which can vary significantly across studies. For example, the “decision” time can be defined from the moment of documentation to team alert or patient preparation, while “incision” may be ambiguous, referring to either the skin or uterine incision [1].

This lack of consensus on terminologies and definitions not only creates potential medico-legal loopholes [40] but may also impede communication among the clinical team and complicate the comparison of obstetric audit or research findings [1,2]. In our study, we addressed these ambiguities by defining each subinterval more precisely, as detailed in the Methods section. Although a distinct subinterval between anesthetic readiness and the first skin incision has been described, we included this phase as part of the anesthetic time, as it is likely negligible in practical terms.

While our findings provide valuable insights for improving obstetric and anesthetic care, the single-center design of the study limits their generalizability. Nevertheless, our findings may still be relevant for other major obstetric centers, given that we operate as Slovenia’s largest tertiary center, handling a substantial number of cesarean deliveries.

Another limitation is the potential misclassification of cesarean sections according to urgency; this decision can be highly individual. Although we provided recommendations for categorizing different urgency groups, the attending obstetrician ultimately made the classification decision, which may lead to significant variability in interpreting the clinical rationale for categorization. Notably, some cases classified as category 3, particularly those with substantially prolonged decision-to-transfer intervals (in five cases exceeding 90 min), may have actually been category 4 urgency.

Since the study aimed to observe and analyze outcomes from routine clinical practice rather than impose a controlled intervention, randomization was not applicable. Instead, the management of each case reflected real-world clinical decision-making processes. Additionally, the observational nature of this study limits our ability to establish causal relationships due to the potential influence of unmeasured confounding factors. While maternal age and BMI did not significantly influence anesthetic choice in our institution, we recognize that the decision-making process for obstetric anesthesia is complex, involving various patient-related, anesthesia-related, obstetric, fetal, and logistical factors. Furthermore, we did not analyze specific events and predisposing factors in each DDI subinterval to better understand their significance. For instance, while some components of the DDI, such as process factors like patient transfer, may be easier to improve, patient-specific factors such as obesity or previous surgeries, which affect delivery time, may pose greater challenges [15,16].

Accordingly, a more comprehensive and standardized assessment of DDI—taking into account the influencing factors for distinct subintervals and their varying susceptibility to improvement—would better facilitate quality improvement efforts for obstetric and anesthetic services.

## 5. Conclusions

Our study highlights the significance of anesthetic choice in category 2 and 3 cesarean deliveries, demonstrating its impact on DDIs and specific maternal-neonatal outcomes. We evaluated three distinct DDI subintervals: transfer time, anesthetic time, and delivery time. Both epidural and general anesthesia were associated with significantly shorter DDIs compared to spinal anesthesia, with over 80% of cases in the former two groups having intervals under 30 min.

Although spinal anesthesia was linked to longer DDIs (which include transfer and anesthetic subintervals), it did not significantly affect the incision to birth or incision to closure durations. Overall, neonatal outcomes were comparable between general and regional anesthetic techniques. The difference in NICU admissions can be attributed to the higher rates of prematurity observed in the general and spinal anesthesia groups. Conversely, maternal blood loss and postoperative complications were significantly greater with general anesthesia compared to neuraxial techniques.

Our findings offer valuable insights for guiding obstetric and anesthetic planning for intrapartum cesarean deliveries. However, further validation through a larger, multi-center study is necessary to draw more definitive conclusions.

## Figures and Tables

**Table 1:** Data Categories and specific data points for all anesthetic groups.

**1. Maternal characteristics:**
Body mass index (BMI)
Parity
Maternal age
Gestational age
Mode of conception: e.g., in vitro fertilization (IVF)
**2. Perioperative time intervals:**
Decision-to-delivery interval (DDI)
Operating room admission to skin incision interval (anesthetic time)
**3. Specific recorded time points:**
Decision to perform a cesarean section
Patient admission into the operating room
First skin incision
Time of delivery (of first fetus in case of multiple gestation)
End of operative procedure (skin closure)
**4. Neonatal outcomes:**
Apgar score at 5 min (good outcome: ≥7)
Birthweight
Umbilical artery pH (good outcome: ≥7.00)
Umbilical artery base excess (good outcome: >−12)
Neonatal intensive care unit (NICU) admission (good outcome: no admission)
Note: For multiple pregnancies, neonatal outcomes were assessed individually.
**5. Maternal outcomes:**
Intraoperative blood loss
Surgical complications (uterine, bladder, or intestinal injury)
Postoperative complications (anemia (Hb < 100 g/L), wound infection, wound hematoma, endometritis)

**Table 2:** Maternal clinical and obstetric characteristics with respect to anesthetic modality.

	General Anesthesia	Spinal Anesthesia	Epidural Anesthesia	*p* Value
Number of patients	89	61	65	
Maternal age (years), mean + SD	31.7 ± 5.7	32.8 + 5.1	31.6 ± 4.6	*p* = 0.327
BMI, mean + SD	25.0 ± 4.8	24.7 ± 5.2	24.7 ± 4.3	*p* = 0.917
Conception by IVF, *n* (%)	6 (6.7%)	5 (8.2%)	6 (9.2%)	*p* = 0.848
Nulliparous, *n* (%)	56 (62.9%)	33 (54.1%)	63 (96.9%)	*p* < 0.001
Multiple pregnancies, *n* (%)	4 (4.5%)	7 (11.5%)	0	*p* = 0.014
Preterm birth <32nd week, *n* (%)	9 (10.1%)	4 (6.6%)	0	*p* = 0.033
Preterm birth 32nd to < 37th week, *n* (%)	15 (16.9%)	15 (24.6%)	0	*p* < 0.001
Term birth ≥ 37th week, *n* (%)	65 (73.0%)	42 (68.9%)	65 (100%)	*p* < 0.001

Note: BMI = body mass index, IVF = in vitro fertilization, SD = standard deviation. Data are presented as mean + standard deviation or *n* (%) and with a significance threshold at *p* < 0.05.

**Table 3:** Perioperative time intervals and maternal-neonatal outcomes for different anesthetic groups.

	General Anesthesia	Spinal Anesthesia	Epidural Anesthesia	*p* Value
DDI (min), mean + SD	25.8 ± 28.8	41.4 ± 26.4	25.1 ± 11.4	*p* < 0.001
DDI ≤ 30 min	75 (84.3%)	26 (42.6%)	56 (86.2%)	*p* < 0.001
Transfer time (min), mean + SD	14.4 ± 28.1	22.5 ± 23.5	11.8 ± 10.8	*p* = 0.024
Anesthetic time (min), mean + SD	7.9 ± 4.0	14.0 ± 5.1	8.9 ± 3.6	*p* < 0.001
Delivery time (min), mean + SD	3.5 ± 3.2	4.9 ± 3.9	4.4 ± 3.7	*p* = 0.055
Surgical procedure time (min), mean + SD	38.4 ± 15.1	35.8 ± 13.3	34.6 ± 9.8	*p* = 0.202
Birthweight (g), mean + SD	2988 ± 909	3049 ± 956	3518 ± 425	*p* < 0.001
Umbilical artery pH < 7	0	0	0	
Umbilical artery pH < 7.10, *n* (%)	2 (2.2%)	0	2 (3.1%)	*p* = 0.416
Base excess, nmol/L, <−12 (%) in Umbilical artery, *n* (%)	2 (2.2%)	0 (1.6%)	2 (3.1%)	*p* = 0.865
5 min Apgar score < 7, *n* (%)	5 (5.6%)	1 (1.6%)	1 (1.6%)	*p* = 0.260
NICU admission, *n* (%)	17 (19.1%)	12 (19.7%)	3 (4.6%)	*p* = 0.021
Blood loss (ml), mean + SD	525 ± 277	473 ± 311	412± 126	*p* = 0.016
Surgical complications, *n* (%)	22 (24.7%)	10 (16.4%)	17 (26.2%)	*p* = 0.363
Postoperative complications, *n* (%)	28 (31.5%)	11 (18.0%)	7 (10.8%)	*p* = 0.006

Note: DDI = decision-to-delivery interval, Transfer time = decision for cesarean delivery to admission to the operation room, Anesthetic time = admission to operating room to first skin incision, Delivery time = skin incision to the delivery of first fetus, Surgical procedure time = skin incision to skin closure, NICU = neonatal intensive care unit, Surgical complications = uterine, bladder, or intestinal injury, Postoperative complications = anemia (Hb < 100 g/L), wound infection, wound hematoma, endometritis, SD = standard deviation. Data are presented as mean + standard deviation or *n* (%) and with a significance threshold at *p* < 0.05.

## Data Availability

The data reported in this study are available upon reasonable request to the corresponding author.
